# Sedation in the intensive care unit with remifentanil/propofol versus midazolam/fentanyl: a randomised, open-label, pharmacoeconomic trial

**DOI:** 10.1186/cc4939

**Published:** 2006-06-15

**Authors:** Bernd Muellejans, Thomas Matthey, Joachim Scholpp, Markus Schill

**Affiliations:** 1 Department of Anaesthesiology and Intensive Care Medicine, Heart Centre Mecklenburg-Vorpommern, Germany; 2 Medical Department, GlaxoSmithKline, Munich, Germany

## Abstract

**Introduction:**

Remifentanil is an opioid with a unique pharmacokinetic profile. Its organ-independent elimination and short context-sensitive half time of 3 to 4 minutes lead to a highly predictable offset of action. We tested the hypothesis that with an analgesia-based sedation regimen with remifentanil and propofol, patients after cardiac surgery reach predefined criteria for discharge from the intensive care unit (ICU) sooner, resulting in shorter duration of time spent in the ICU, compared to a conventional regimen consisting of midazolam and fentanyl. In addition, the two regimens were compared regarding their costs.

**Methods:**

In this prospective, open-label, randomised, single-centre study, a total of 80 patients (18 to 75 years old), who had undergone cardiac surgery, were postoperatively assigned to one of two treatment regimens for sedation in the ICU for 12 to 72 hours. Patients in the remifentanil/propofol group received remifentanil (6- max. 60 μg kg^-1 ^h^-1^; dose exceeds recommended labelling). Propofol (0.5 to 4.0 mg kg^-1 ^h^-1^) was supplemented only in the case of insufficient sedation at maximal remifentanil dose. Patients in the midazolam/fentanyl group received midazolam (0.02 to 0.2 mg kg^-1 ^h^-1^) and fentanyl (1.0 to 7.0 μg kg^-1 ^h^-1^). For treatment of pain after extubation, both groups received morphine and/or non-opioid analgesics.

**Results:**

The time intervals (mean values ± standard deviation) from arrival at the ICU until extubation (20.7 ± 5.2 hours versus 24.2 h ± 7.0 hours) and from arrival until eligible discharge from the ICU (46.1 ± 22.0 hours versus 62.4 ± 27.2 hours) were significantly (*p *< 0.05) shorter in the remifentanil/propofol group. Overall costs of the ICU stay per patient were equal (approximately €1,700 on average).

**Conclusion:**

Compared with midazolam/fentanyl, a remifentanil-based regimen for analgesia and sedation supplemented with propofol significantly reduced the time on mechanical ventilation and allowed earlier discharge from the ICU, at equal overall costs.

## Introduction

In western industrialised countries, a substantial amount of the gross domestic product is spent on health care; for example, almost 11% in Germany. About 30% of the health care expenditure is caused by inpatient curative and rehabilitative care [1]. Intensive care units (ICUs) are among the costliest areas of the hospital. Personnel costs account for an estimated 35% to 60% of the ICU budget and pharmacy costs for 10% to 23%. Sedatives and analgesics only constitute approximately 1% to 3.5% of the ICU costs [2].

The major goals of analgesia and sedation for critically ill patients in the ICU are to provide control of pain and anxiolysis and to facilitate mechanical ventilation and therapeutic and diagnostic interventions. Patients should be easily arousable, calm and co-operative [3]. Mostly, a combination of an opioid, such as fentanyl, sufentanil or morphine, to provide analgesia and a benzodiazepine or propofol to provide sedation is used.

When administered over several hours or even days, however, elimination of most drugs for analgesia and sedation may be prolonged in critically ill patients as a result of accumulation due to organ-dependent elimination. This can result in delayed emergence from sedation after discontinuation of administration, increased time on the ventilator and in the ICU and, therefore, increased costs [4]. Moreover, prolonged sedation may have not only economic but also medical consequences, such as failure to recognize cerebral insult, immunosuppression or venous stasis, which may promote thromboembolism [4,5].

Remifentanil hydrochloride is a potent, selective μ-opioid receptor agonist, indicated for the provision of analgesia in mechanically ventilated critically ill patients for up to three days. Its organ-independent elimination and short context-sensitive half time of 3 to 4 minutes lead to a highly predictable offset of action [6].

These properties make remifentanil a useful analgesic in critically ill patients requiring analgesia and sedation. Several studies have been published describing the potential role and actual use of remifentanil in the ICU [7-10]. Several recently published studies showed that the use of remifentanil can result in shorter time to extubation in comparison to morphine [11,12], fentanyl [12] and sufentanil [13]. From a pharmacological view, propofol is the best concomitant sedative for remifentanil, as it leads to shorter awakening times than midazolam [14]. Recently published German guidelines recommend the use of short acting drugs for analgesia and sedation for less than 24 hours [15].

In our study, midazolam/fentanyl was chosen as the comparator-regimen for two reasons: first, with regard to the direct drug costs, it is the cheapest alternative; and second, it is the most widely used regimen in German ICUs [16]. Cardiac surgical patients were selected because the ICU bed is often the 'bottleneck' leading to postponement of surgical procedures. Speeding the recovery process would lead to a higher turnover of patients, reduced costs per patient, less postponed operations and to a more efficient use of ICU resources.

This clinical and cost-consequence study was designed to compare a remifentanil-based sedation regimen supplemented with propofol with a conventional midazolam/fentanyl regimen in patients after cardiac surgery requiring postoperative mechanical ventilation in the ICU.

## Materials and methods

This randomised, open-label, single-centre, parallel group study was conducted in accordance with good clinical practice and with the guidelines set out in the Declaration of Helsinki. After local ethics committee approval, a total of 80 patients were recruited. Preoperatively, informed consent was obtained from all patients.

Patients who had undergone elective coronary artery and/or heart valve surgery were eligible for entry into the study if they were aged 18 to 75 years, were intubated and were expected to require mechanical ventilation for 12 to 72 hours.

Patients were excluded from the study if one of the following conditions was given or expected to become applicable: pre-existing impaired central nervous system function, weight >120 kg, use of neuromuscular blocking agents in the ICU, epidural anaesthesia, ASA (American Society of Anesthesiologists) IV and V. Patients with a history of allergy to study medication or of opioid abuse were also excluded from the study. Patients who required analgesia and sedation beyond 72 hours or a tracheostomy and pregnant or lactating women were excluded from the study.

Double blinding was judged to be impractical due to the different dosing algorithms and physical characteristics of the drugs used. Furthermore, in a double-blinded study the medical staff would most probably have recognized the regimen at discontinuation of study drugs due to their diverging pharmacokinetic properties.

Anaesthesia for cardiac surgery was performed in both groups according to routine practice in the study centre, with a total intravenous technique consisting of remifentanil, propofol, clonidine and cisatracurium. After termination of the operation, remifentanil was continued at the investigator's discretion to maintain analgesia during the transfer of patients to the ICU. The propofol infusion was stopped on arrival at the ICU at the latest.

After arrival in the ICU, patients were randomised on a 1:1 basis to receive either remifentanil (*n *= 40), titrated to provide optimal analgesia and supplemented with propofol if additional sedation was required, or a conventional treatment regimen consisting of midazolam and fentanyl (*n *= 40), administered simultaneously and then titrated to response. The doses of propofol, midazolam and fentanyl were in accordance with the guidelines of the Society of Critical Care Medicine [14]. The maximum remifentanil dose was higher than the maximum dose recommended in the SPC to provide effective analgesia-based sedation in this group.

According to the clinic's usual practice, the level of sedation was judged according to a simple three-step sedation score (Table 1) and the dosing of the sedative agents was adapted accordingly.

### Treatment protocols

#### Remifentanil-based analgesia and sedation

On arrival on the ICU, the remifentanil infusion was continued or started at an initial rate of 6 to 12 μg kg^-1 ^h^-1 ^and was increased depending on clinical need up to a maximum of 60 μg kg^-1 ^h^-1^. If an adequate level of sedation was not achieved with remifentanil alone at an infusion rate of 60 μg kg^-1 ^h^-1^, additional sedation was provided by administering a bolus dose of propofol (0.3 to 1.0 mg kg^-1^) and/or a propofol infusion starting at a rate of 0.5 to 1.0 mg kg^-1 ^h^-1^. If the adequate level of sedation was still not achieved, the patient received additional boluses and/or increases in the infusion rate of propofol up to a maximum dose of 4 mg kg^-1 ^h^-1^. In the case of excessive sedation, the propofol infusion rate was reduced first.

#### Hypnotic-based sedation with midazolam/fentanyl

At arrival on the ICU, patients received an initial bolus dose of fentanyl of 1 to 2 μg kg^-1^, followed by an infusion at an initial rate of 1 to 2 μg kg^-1 ^h^-1^. Additionally, all patients received an initial bolus dose of midazolam. Although this bolus could range from 0.03 to 0.2 mg kg^-1^, patients commonly received a 2 mg bolus at the lower end of this range. This was followed by an infusion at an initial rate of 0.02 to 0.04 mg kg^-1 ^h^-1^.

In case of insufficient sedation, additional bolus doses of midazolam were given and/or the midazolam infusion was increased up to a maximum of 0.2 mg kg^-1 ^h^-1^. In the case of insufficient analgesia, fentanyl was increased up to a maximum of 7 μg kg^-1 ^h^-1^.

### Weaning, extubation and discharge

Only patients who were expected to require mechanical ventilation for at least 12 hours were included in the study. The weaning process was started on the morning after the day of the operation at 0700 hours if no surgical complication was anticipated, if there were no signs of respiratory or haemodynamic impairment or acute organ insufficiency and if the rectal body temperature was >36.5°C. When weaning was started, all infusions were stopped and patients in the remifentanil/propofol group received a bolus of morphine (0.1 to 0.3 mg/kg). For treatment of pain after extubation, both groups received morphine (a bolus of 0.1 to 0.3 mg/kg) and/or other analgesics, at the investigator's discretion. Extubation was performed if there were no signs of major respiratory (tidal volume >4 ml/kg, respiratory rate 10 to 25/minute, p_a_O_2 _(partial pressure of oxygen in arterial blood)>69 mmHg, p_a_CO_2 _(partial pressure of carbon dioxide in arterial blood)<55 mmHg, F_I_O_2 _(fraction of inspired oxygen)<0.5) or of haemodynamic impairment and if the patient was able to follow commands.

Discharge from the ICU was performed if there were no signs of neurological (Ramsay sedation score 2, co-operative, oriented, tranquil), respiratory (p_a_O_2 _>69 mmHg, p_a_CO_2 _= 35 to 45 mmHg, inspired O_2 _<3 l/minute), haemodynamic (no catecholamines, no significant fluid deficit) or surgical (no anticipated surgical complication) impairment and if the pain score on the visual analogue scale (VAS) was <4.

### Efficacy assessment points

**Figure 1:**
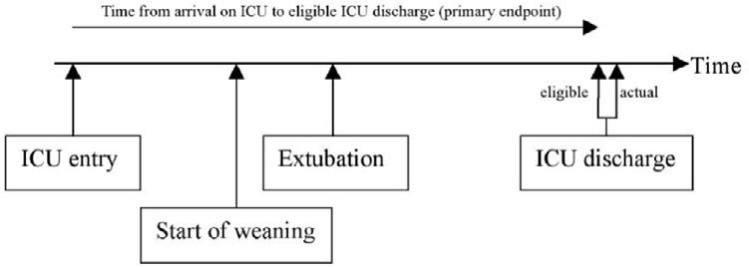
Recorded time points from arrival on the intensive care unit to discharge.

To assess the efficacy of the two regimens, several time points were recorded throughout the treatment period (Figure 1). The time to the start of the weaning procedure was defined as the time from arrival on the ICU to the first time the study drug was reduced in order to encourage spontaneous respiration, which subsequently led to extubation. The weaning time was recorded, calculated as the time interval from start of the weaning until extubation. Finally, time intervals from arrival on the ICU until extubation and eligibility for (primary end point) and actual discharge from ICU were recorded.

### Safety

Adverse events, defined as any untoward medical occurrence in a patient administered a pharmaceutical product and that does not necessarily have a causal relationship with this treatment, were recorded from the start of the study drug until discharge from ICU. Serious adverse events were defined as adverse events that resulted in any of the following outcomes: death, life-threatening event, prolongation of hospitalisation, or a disability or incapacity. Important medical events that did not result in death or were not life-threatening were also considered serious adverse events when, on the basis of appropriate medical judgement, they jeopardised the patient and required medical or surgical intervention to prevent one of the outcomes listed above.

### Cost calculation

Drug costs (including all concomitant medication and wastage), costs of materials for analgo-sedation (only variable costs), and personnel costs in the ICU were considered. All other types of costs were assumed to not significantly differ between regimens. Indirect costs (productivity loss) were excluded. The resource utilisation was derived from the study centre. Generic unit costs (price level 2003) based on publicly available databases [17-19] were applied for resource valuation. The financial department of the study centre checked these unit costs to ensure that they represent realistic estimates.

Official ex-factory prices were used for the calculation of drug costs (e.g., costs of study drugs: remifentanil €5.36/mg, propofol €19.34/g, fentanyl €5.42/mg and midazolam €0.124/mg) [17]. For the costs of blood products official hospital tariffs [18] were applied, while for the costs of materials the average unit costs from two hospitals and two internet shops were taken. Personnel costs on the ICU were calculated based on the times measured in the study and on cost rates per hour and patient, which were different for physicians and nurses. The cost rates are based on specific, real-world cost rates from a German 400 bed hospital [19] that were extrapolated to 2003 [20,21]. The resulting cost rates were €6.19 or €15.68 per hour of care per patient for physicians and nurses, respectively. As personnel costs vary during ICU stay depending on the intensity of care, the ICU stay was divided into three periods (ventilation period (including weaning), treatment period (intensive care activities other than ventilation), and monitoring only period), and the respective personnel-cost-multipliers were applied (1.00, 1.00, 0.67, respectively, for physicians; 1.71, 1.00, 0.57, respectively, for nurses) according to the calculation of German Diagnosis Related Groups [22].

### Statistics

At the planning state of this study only very limited data regarding the expected difference between the two groups and their variability had been published with regard to the primary endpoint. Therefore, a two-stage adaptive study design according to Bauer and Köhne [23] was used. Based on an interim analysis with 30 patients, standard deviation and effect size were estimated. Based on these data, it was determined by the planning statistician to recruit at least 40 additional patients in the second stage. If all patients of the second stage were evaluable for the primary efficacy variable, the total sample size should guarantee a global power of at least 80% for a standardised effect size of 0.7, which was deemed a reasonable setting according to the interim analysis. Due to the loss of power when using Fisher's combination of p values as the global test, formally a power of 83% and an adjusted alpha had to be used for sample size estimation for the second stage. To show superiority of the remifentanil regimen in the final analysis at the 5% significance level, the product of two one-sided *p *values, associated with the respective *t *tests for the two parts of the study, was compared with a critical value of 0.0087 [23].

This combination test was used for the primary efficacy variable only. For all other tests the interim analysis did not lead to further adjustments.

Adverse event rates were compared by Fisher's exact test. Length of time periods and costs were analysed by means of t-tests. For ordinal data (like the simplified acute physiology score (SAPS)) the Wilcoxon rank-sum test was used. As usual, all secondary statistical tests should be interpreted in a descriptive manner only.

### Scenario and sensitivity analysis

A decision analysis model representing the study was built. It was employed to simulate the results gained with a different remifentanil/propofol regimen and to explore the robustness of the results for modelling parameter variations.

## Results

A total of 80 patients were enrolled in the study, of which 72 could be evaluated (modified intent-to-treat population). Of these, 8 patients (7 patients in the midazolam/fentanyl and 1 patient in the remifentanil/propofol group) had to be excluded during the study: 3 patients due to mechanical ventilation >72 hours, 4 patients due to reintubation and 1 patient because he was only randomised but did not receive study medication due to postoperative bleeding. The seven treated patients were excluded because the primary efficacy measure could not be assessed due to a lack of essential data. Demographic and baseline characteristics are shown in Table 2. Apart from a higher SAPS II on admission in the remifentanil/propofol group (*p *< 0.05, Wilcoxon rank-sum test), patients were well matched in the two treatment groups.

Table 3 presents the incidence of adverse events. There was no statistically significant difference between the remifentanil/propofol group and the midazolam/fentanyl group in terms of the overall number of subjects with adverse events (23 versus 24 patients) or serious adverse events (4 versus 6 patients). Significantly more patients in the remifentanil/propofol group than in the midazolam/fentanyl group suffered from drug-related adverse events.

Similarly, the mean percentage time of adequate, inadequate or excessive sedation between the remifentanil/propofol and the midazolam/fentanyl group did not differ statistically significantly (59% versus 70%, 13% versus 11% and 28% versus 19%, respectively) when compared by means of the *t *test.

Propofol was added in 21 (54%) of the 39 patients receiving remifentanil. The mean infusion rates including bolus doses were 41.2 μg kg^-1 ^h^-1 ^for remifentanil (bolus doses were not allowed), 2.2 mg kg^-1 ^h^-1 ^for propofol in patients receiving propofol (which results in a mean of 1.2 kg^-1 ^h^-1 ^propofol for all patients in the remifentanil/propofol group), 0.06 mg kg^-1 ^h^-1 ^for midazolam and 3.8 μg kg^-1 ^h^-1 ^for fentanyl.

Table 4 presents the time intervals from arrival on ICU to assessment points and Table 5 shows the costs by category.

Because of the study protocol, the used remifentanil doses (equivalent to baseline) were higher than in routine clinical practice. To estimate the costs under routine circumstances, we performed a scenario analysis. We lowered the mean remifentanil infusion rate from 41.2 μg kg^-1 ^h^-1 ^to 9 μg kg^-1 ^h^-1^, increased the propofol infusion rate from 1.2 mg kg^-1 ^h^-1 ^to 4 mg kg^-1 ^h^-1 ^and assumed that this routine practice scenario would have rendered the same reduction (24%) in personnel costs compared to the midazolam/fentanyl regimen and identical material and drug use (without study drugs) as the baseline. This scenario led to 56% lower remifentanil/propofol drug costs or net savings of €214 per patient when compared to baseline or to the midazolam/fentanyl regimen, respectively. In addition, the model analysis showed that with the routine remifentanil/propofol doses, even a personnel cost reduction of 10% would be sufficient to render cost savings. The univariate sensitivity analysis demonstrated that variations in nurse and study drug costs might have a strong impact on our cost results.

## Discussion

Sedation on the ICU can be either analgesia or sedative based. With a sedative-based regimen, hypnotic agents are titrated to maintain patient comfort despite them having almost no analgesic effect, and the opioid dose is usually minimised. Patients are therefore kept asleep but are not necessarily pain free. When interviewed about their ICU stay, many patients recall significant unrelieved pain [24-26]. Pain may evoke a stress response leading to adverse effects such as tachycardia, increased myocardial oxygen consumption, hypercoagulability, immunosuppression and persistent catabolism [14,27]. Moreover, a sedative-based regimen may facilitate oversedation, which may lead to prolonged mechanical ventilation and longer stays in both the ICU and hospital [4]. The increased duration of mechanical ventilation may translate into nosocomial complications, such as ventilator-associated pneumonia [28]. Over-sedation may impede a recommended [29] daily interruption or lightening of sedation, increase the incidence of complications [5], hinder neurological assessment and increase costs through the need for a greater number of expensive tests such as CT scans of the brain [30].

The aim of analgesia-based sedation is to focus in the first instance on achieving effective analgesia, with a sedative agent being given subsequently if required. Effective analgesia may diminish the stress response, provide comfort, and facilitate treatment of critically ill septic patients. Guidelines recommend that sedation of critically ill patients should be started only after providing adequate analgesia [14,15,31,32].

The ideal sedative agent should be effective and easily titratable, with a rapid onset and offset of action, no accumulation, and it should be cost-effective by improving the quality of care, reducing the time spent on mechanical ventilation or reducing the length of stay in the ICU [4]. Except for higher acquisition costs, remifentanil fulfils these attributes.

We have shown that after cardiac surgery, analgesia-based sedation with remifentanil and propofol allows a facilitated turnover of patients, achieved by significantly earlier extubation and discharge from the ICU, and can be administered at equal total costs, compared with a conventional sedation regimen with midazolam and fentanyl. Although both remifentanil and propofol are considerably more expensive than midazolam and fentanyl, the cost savings achieved by a shorter weaning time, leading to earlier extubation and an earlier discharge from the ICU, outweigh the higher acquisition costs. No difference between the two groups in the level of sedation or in safety was observed.

The shorter weaning time with remifentanil/propofol is a direct consequence of the pharmacokinetic profile of these two drugs. Remifentanil has a rapid onset (1 minute) and offset (half time <10 minutes) of action [33]. Its organ-independent metabolism by non-specific blood and tissue esterases results in a pharmacokinetic profile unaffected by impaired kidney [9,34] or liver [35] function, which differentiates remifentanil from all other opioids. Remifentanil does not accumulate, even after prolonged infusion [36,37].

Although both midazolam and fentanyl have a rapid onset and a short clinical duration with single doses, accumulation and prolonged sedative effects may be observed after continuous administration [38], which is also indicated by a significantly longer context-sensitive half time of these drugs [36,39].

Time from extubation (after discontinuation of the study medication) until discharge from the ICU was also significantly longer in the midazolam/fentanyl group. This recovery period after a long and difficult operation, such as open heart surgery with cardiopulmonary bypass in elderly patients with multiple co-morbidities, certainly is a multi-factorial event. Although patients in the remifentanil/propofol group were more severely ill, as shown by a higher SAPS II, a difference in vigilance, orientation and compliance after extubation was obvious. Patients in the midazolam/fentanyl group reached the predefined discharge criteria later. An accumulation of sedatives most likely can be presumed to be the reason.

With respect to adverse and serious adverse events and the duration of adequate sedation, no statistically significant differences could be determined. These findings correspond well with the published literature [12]. The higher incidence of drug-related adverse events in the remifentanil/propofol group, mainly consisting of shivering, might be due to the unusually high remifentanil dose in our study and is not consistent with the findings of other studies [8,12].

As pointed out, the mean infusion rate for remifentanil was very high (41.2 μg kg^-1 ^h^-1^), leading to very high drug costs. The summary of product characteristics of remifentanil recommends a starting dose of 6 to 9 μg kg^-1 ^h^-1 ^and the addition of a sedative drug already at a rate of 12 μg kg^-1 ^h^-1^. In our study, in spite of a very high remifentanil infusion rate of 60 μg kg^-1 ^h^-1^, more than half of the patients still needed the addition of propofol. Presumably, the earlier addition of propofol, following the recommendations in the summary of product characteristics of remifentanil, would have considerably reduced the drug costs of remifentanil, with only a smaller increase in the costs for propofol. As demonstrated in our scenario analysis, this real world scenario might even render cost savings to the hospital. This assumption is further supported by a recently published study in which the mean remifentanil infusion rate was 7.8 μg kg^-1 ^h^-1^, with a remifentanil 'trigger' dose for the addition of midazolam of 12 μg kg^-1 ^h^-1 ^[11]. In this study, extubation could be performed within 17 minutes after a duration of mechanical ventilation of more than 14 hours.

In addition to this cost-reducing consideration, according to the study protocol, a high concentration of 250 μg/ml remifentanil in the infusion syringes (10 mg remifentanil in 40 ml infusion solution) led to high wastage costs of remifentanil, which are included in the drug costs. A lower concentration certainly would reduce wastage costs and should be recommended in short term sedation.

Our study shows several limitations: as in any open study, there is the risk of biased patient assessment and treatment. On the other hand, an open study design offers the opportunity to investigate the drugs under real world conditions, that is, it measures the effectiveness instead of the efficacy.

When the protocol was being designed, we felt that it might be more feasible to use a three point scale instead of a validated sedation scale like the Sedation-Agitation Scale (SAS) or the Richmond Agitation Sedation Scale (RASS) for evaluating sedation, or the Visual Analogue Scale (VAS) or the Behavioural Pain Scale (BPS) for evaluating pain. To date, we would also prefer to use these validated scales.

Concomitant medications were not limited and not recorded, and data on tolerance and withdrawal were not collected in our trial.

According to standardised processes of the hospital, weaning was started on the morning after the operation at 0700. This was fixed due to an established one-shift system for physicians in the study centre and ensured close assessments in regard to extubation and discharge criteria during the daytime. This procedure led to longer postoperative mechanical ventilation and sedation than a fast track regimen, resulting in a mean time to weaning of more than 18 hours in both groups. A three-shift system would likely have enabled an earlier weaning, which might have had impact on time to extubation and discharge in both groups. However, the applied procedure resulted in the positive side effect of similar mechanical ventilation times in both groups and thus high comparability of the investigated regimens.

Finally, as our study was conducted in one German hospital, its results might not be directly transferable to other countries and settings. Instead, a case-by-case check of the transferability is advised.

## Conclusion

We conclude that analgesia and sedation with remifentanil and propofol can facilitate a higher turnover of patients by reducing time on mechanical ventilation and by shortening the overall length of ICU stay compared to a conventional regimen with midazolam and fentanyl. Higher drug costs of remifentanil and propofol are compensated by reduced personnel costs on the basis of earlier discharge and may even lead to cost-savings to the hospital, depending on the dosing algorithm.

Remifentanil and propofol are useful tools for analgesia and sedation during the weaning phase and for short to medium term sedation in the ICU.

## Key messages

• 	Remifentanil based analgesia and sedation was shown to be effective and well tolerated in postoperatively ventilated patients who had undergone cardiac surgery

• 	A remifentanil/propofol regimen compared with a midazolam/fentanyl regimen may: reduce the time on mechanical ventilation; shorten the ICU stay; and be cost-neutral or even lead to cost-savings in the short to medium term mechanically ventilated patients, depending on the setting and dosing algorithm

## Abbreviations

ASA = American Society of Anesthesiologists; BPS = Behavioural Pain Scale; F_i_O_2 _= fraction of inspired oxygen; ICU = intensive care unit; paCO_2 _= partial pressure of carbon dioxide in arterial blood; paO_2 _= partial pressure of oxygen in arterial blood; SAPS = simplified acute physiology score; SPC = summary of product characteristics; RASS = Richmond Agitation Sedation Scale; VAS = Visual Analogue Scale.

## Competing interests

BM and TM received payment from GlaxoSmithKline (either personally or to their respective department), depending on the number of patients recruited. JS and MS are employees of GlaxoSmithKline.

## Authors' contributions

JS made substantial contributions to the conception and design of this study and provided critical review of the manuscript. BM and TM performed the study and provided critical review of the manuscript. BM and MS drafted the manuscript. All authors read and approved the manuscript.

## Figures and Tables

**Table 1:** Three-step sedation score

Step	Level of sedation	Definition
1	Insufficient	Patient moves or grimaces without stimuli, patient is coughing or lacrimates
2	Adequate	Patient is calm, moves on stimuli
3	Excessive	Patient does not react to stimuli

**Table 2:** Patient demography and baseline characteristics

	Remifentanil/propofol	Midazolam/fentanyl
Number of patients analysed	39	33
Mean age (years)	65 ± 8.1	66.5 ± 7.0
Male/female	30/9	23/10
Mean height (cm)/weight (kg)	169.9 ± 8.8/80.1 ± 12.7	170.5 ± 7.1/83.9 ± 12.1
ASA physical status (I/II/III)	0/0/39	0/0/33
Type of surgery^a^		
Bypass surgery	33	31
Valve surgery	11	8
Mean ejection fraction (%) at screening	49.5 ± 12.7	50.3 ± 15.8
Mean SAPS II on admission	37.6 ± 14.2	28.2 ± 11.7

Values are number of patients or mean ± standard deviation. ^a^Multiple counts are possible per subject. SAPS, simplified acute physiology score.

**Table 3:** Adverse event profile

	Remifentanil/propofol (*n *= 40)	Midazolam/fentanyl (*n *= 39)	*P *value^a^
Any adverse event	23	24	NS
Any drug-related adverse event	9	2	<0.05
Shivering	8	2	NS
Pain	2	0	NS
Any serious adverse event	4	6	NS
Any drug-related serious adverse event	0	0	NS
Most commonly occurring adverse events (≥ 5% of patients)			
Shivering	14	6	NS
Delirium	7	9	NS
Pain	3	0	NS
Tachycardia	2	0	NS
Neuropathy (nerve injury, e.g., by compression)	1	2	NS
Respiratory insufficiency	0	3	NS
Hypotension	3	0	NS
Haemorrhage	2	2	NS

Values are number of patients with event; multiple entries per patient possible.

^a^Fisher's exact test, two tails, significance level 0.05. NS, not significant.

**Table 4:** Time assessment points

Time assessment point (hours)	Remifentanil/propofol (*n *= 39)	Midazolam/fentanyl (*n *= 33)	*P *value^a^
Time from arrival on ICU to weaning	18.5 ± 2.5	18.5 ± 2.2	NS
Weaning time	2.2 ± 4.3	5.7 ± 6.6	<0.05
Time from arrival on ICU to extubation	20.7 ± 5.2	24.2 ± 7.0	<0.05
Time from extubation to eligible ICU discharge	25.4 ± 19.7	38.2 ± 26.3	<0.05
Time from arrival on ICU to eligible ICU discharge^b^	46.1 ± 22.0	62.4 ± 27.2	<0.05
Time from arrival on ICU to actual ICU discharge	46.4 ± 21.9	64.7 ± 29.3	<0.05

Values are means ± standard deviation. ^a^*t *test. ^b^Primary endpoint. ICU, intensive care unit; NS, not significant.

**Table 5:** Overall costs (€, 2003 price level) of the intensive care unit stay per patient

	Remifentanil/propofol (*n *= 39)	Midazolam/fentanyl (*n *= 33)	*P *value^a^
Material	11/10 (5–25)	11/11 (4–18)	NS
Non-study drugs	218/137 (31–1257)	192/153 (55–723)	NS
Study drugs	353/341 (54–835)	42/46 (19–63)	<0.05
Nurses	869/827 (517–2213)	1,131/1,071 (554–2,164)	<0.05
Physicians	260/243 (135–723)	352/301 (151–811)	<0.05
Overall	1,712/1,558 (1,058–3,647)	1,729/1,604 (821–3,337)	NS

Values are means/medians (range). ^a^Wilcoxon rank-sum test. NS, not significant.

## References

[B1] Organisation for Economic Co-operation and Development (OECD) (2004). OECD Health Data 2004.

[B2] Cheng EY (1995). The cost of sedating and paralyzing the critically ill patient. Crit Care Clin.

[B3] Tonner PH, Weiler N (2003). Sedation and analgesia in the intensive care unit. Curr Opin Anaesthesiol.

[B4] Ramsey MAE (2000). Intensive Care: problems of over- and undersedation. Clin Anaesthesiol.

[B5] Schweickert WD, Gehlbach BK, Pohlman AS, Hall JB, Kress JP (2004). Daily interruption of sedative infusions and complications of critical illness in mechanically ventilated patients. Crit Care Med.

[B6] Scott LJ, Perry CM (2005). Remifentanil: a review of its use during the induction and maintenance of general anaesthesia. Drugs.

[B7] Wilhelm W, Dorscheid E, Schlaich N, Niederprum P, Deller D (1999). [The use of remifentanil in critically ill patients. Clinical findings and early experience]. Anaesthesist.

[B8] Muellejans B, Lopez A, Cross MH, Bonome C, Morrison L, Kirkham AJ (2004). Remifentanil versus fentanyl for analgesia based sedation to provide patient comfort in the intensive care unit: a randomized, double-blind controlled trial [ISRCTN43755713]. Crit Care.

[B9] Breen D, Wilmer A, Bodenham A, Bach V, Bonde J, Kessler P, Albrecht S, Shaikh S (2004). Offset of pharmacodynamic effects and safety of remifentanil in intensive care unit patients with various degrees of renal impairment. Crit Care.

[B10] Karabinis A, Mandragos K, Stergiopoulos S, Komnos A, Soukup J, Speelberg B, Kirkham AJ (2004). Safety and efficacy of analgesia-based sedation with remifentanil versus standard hypnotic-based regimens in intensive care unit patients with brain injuries: a randomised, controlled trial [ISRCTN50308308]. Crit Care.

[B11] Dahaba AA, Grabner T, Rehak PH, List WF, Metzler H (2004). Remifentanil versus morphine analgesia and sedation for mechanically ventilated critically ill patients: a randomized double blind study. Anesthesiology.

[B12] Breen D, Karabinis A, Malbrain M, Morais R, Albrecht S, Jarnvig IL, Parkinson P, Kirkham AJ (2005). Decreased duration of mechanical ventilation when comparing analgesia-based sedation using remifentanil with standard hypnotic-based sedation for up to 10 days in intensive care unit patients: a randomised trial [ISRCTN47583497]. Crit Care.

[B13] Baillard C, Cohen Y, Le Toumelin P, Karoubi P, Hoang P, Ait KF, Cupa M, Fosse JP (2005). [Remifentanil-midazolam compared to sufentanil-midazolam for ICU long-term sedation]. Ann Fr Anesth Reanim.

[B14] Jacobi J, Fraser GL, Coursin DB, Riker RR, Fontaine D, Wittbrodt ET, Chalfin DB, Masica MF, Bjerke HS, Coplin WM (2002). Clinical practice guidelines for the sustained use of sedatives and analgesics in the critically ill adult. Crit Care Med.

[B15] Martin J, Bäsell K, Bürkle H, Hommel J, Huth G, Kessler P, Kretz FJ, Putensen Ch, Quintel M, Tonner P (2005). Analgesie und Sedierung in der Intensivmedizin – Kurzversion. Anästhesiol Intensivmed.

[B16] Soliman HM, Mélot C, Vincent JL (2001). Sedative and analgesic practice in the intensive care unit: the results of a European survey. Br J Anaesth.

[B17] Lauer-Taxe online. http://www.lauer-fischer.de.

[B18] Deutsche Krankenhausgesellschaft (DKG) (2002). DKG-NT Band I.

[B19] Bundesministerium für Gesundheit (1995). Leitfaden zur Einführung von Fallpauschalen und Sonderentgelten gemäß Bundespflegesatzverordnung 1995.

[B20] Rolland S, Rosenow C, Klauber J, Robra BP, Schellschmidt H (2005). Statistische Krankenhausdaten: Grund- und Kostendaten der Krankenhäuser 2002. Krankenhaus-Report 2004.

[B21] Statistisches Bundesamt (2004). Statistisches Jahrbuch für die Bundesrepublik Deutschland 2004.

[B22] Deutsche Krankenhausgesellschaft (DKG) (2002). Kalkulation von Fallkosten Handbuch zur Anwendung in Krankenhäusern Version 20.

[B23] Bauer P, Kohne K (1994). Evaluation of experiments with adaptive interim analyses. Biometrics.

[B24] Novaes MA, Knobel E, Bork AM, Pavao OF, Nogueira-Martins LA, Ferraz MB (1999). Stressors in ICU: perception of the patient, relatives and health care team. Intensive Care Med.

[B25] Carroll KC, Atkins PJ, Herold GR, Mlcek CA, Shively M, Clopton P, Glaser DN (1999). Pain assessment and management in critically ill postoperative and trauma patients: a multisite study. Am J Crit Care.

[B26] Ferguson J, Gilroy D, Puntillo K (1997). Dimensions of pain and analgesic administration associated with coronary artery bypass grafting in an Australian intensive care unit. J Adv Nurs.

[B27] Vender JS, Szokol JW, Murphy GS, Nitsun M (2004). Sedation, analgesia, and neuromuscular blockade in sepsis: an evidence-based review. Crit Care Med.

[B28] Cook DJ, Walter SD, Cook RJ, Griffith LE, Guyatt GH, Leasa D, Jaeschke RZ, Brun-Buisson C (1998). Incidence of and risk factors for ventilator-associated pneumonia in critically ill patients. Ann Intern Med.

[B29] Dellinger RP, Carlet JM, Masur H, Gerlach H, Calandra T, Cohen J, Gea-Banacloche J, Keh D, Marshall JC, Parker MM (2004). Surviving Sepsis Campaign guidelines for management of severe sepsis and septic shock. Crit Care Med.

[B30] Wilhelm W, Wrobel M, Ketter R, Steudel W, Kreuer S (2004). Remifentanil/propofol versus fentanyl/midazolam for ICU sedation. Eur J Anaesthesiol.

[B31] Young C, Knudsen N, Hilton A, Reves JG (2000). Sedation in the intensive care unit. Crit Care Med.

[B32] Lane M, Cadman B, Park G (2002). Learning to use remifentanil routinely in the critically ill. Care Critically Ill.

[B33] Westmoreland CL, Hoke JF, Sebel PS, Hug CC, Muir KT (1993). Pharmacokinetics of remifentanil (GI87084B) and its major metabolite (GI90291) in patients undergoing elective inpatient surgery. Anesthesiology.

[B34] Pitsiu M, Wilmer A, Bodenham A, Breen D, Bach V, Bonde J, Kessler P, Albrecht S, Fisher G, Kirkham A (2004). Pharmacokinetics of remifentanil and its major metabolite, remifentanil acid, in ICU patients with renal impairment. Br J Anaesth.

[B35] Navapurkar VU, Archer S, Gupta SK, Muir KT, Frazer N, Park GR (1998). Metabolism of remifentanil during liver transplantation. Br J Anaesth.

[B36] Egan TD, Lemmens HJ, Fiset P, Hermann DJ, Muir KT, Stanski DR, Shafer SL (1993). The pharmacokinetics of the new short-acting opioid remifentanil (GI87084B) in healthy adult male volunteers. Anesthesiology.

[B37] Malbrain M, Karabinis A, Morais R, Albrecht S, Breen D, Parkinson P (2004). Decreased time on mechanical ventilation using remifentanil-based analgesia and sedation. Crit Care.

[B38] Malacrida R, Fritz ME, Suter P, Crevoisier C (1992). Pharmacokinetics of midazolam administered by continuous infusion to intensive care patients. Crit Care Med.

[B39] Hughes MA, Glass PS, Jacobs JR (1992). Context-sensitive half-time in multicompartment pharmacokinetic models for intravenous anesthetic drugs. Anesthesiology.

